# Genome Assembly of Citrus Leprosis Virus Nuclear Type Reveals a Close Association with Orchid Fleck Virus

**DOI:** 10.1128/genomeA.00519-13

**Published:** 2013-07-25

**Authors:** Avijit Roy, Andrew Stone, Gabriel Otero-Colina, Gang Wei, Nandlal Choudhary, Diann Achor, Jonathan Shao, Laurene Levy, Mark K. Nakhla, Charla R. Hollingsworth, John S. Hartung, William L. Schneider, Ronald H. Brlansky

**Affiliations:** University of Florida, IFAS, Plant Pathology Department, Citrus Research and Education Center, Lake Alfred, Florida, USAa; USDA-ARS, FDWSRU, Ft. Detrick, Maryland, USAb; Colegio de Postgraduados, Campus Montecillo, Texcoco, Estado de Mexico, Mexicoc; USDA-APHIS-PPQ-CPHST, Beltsville, Maryland, USAd; USDA-ARS, MPPL, Beltsville, Maryland, USAe; USDA-APHIS-PPQ-CPHST, Riverdale, Maryland, USAf; USDA-APHIS-PPQ-CPHST, Raleigh, North Carolina, USAg

## Abstract

The complete genome of citrus leprosis virus nuclear type (CiLV-N) was identified by small RNA sequencing utilizing leprosis-affected citrus samples collected from the state of Querétaro, Mexico. The nucleotide identity and phylogenetic analysis indicate that CiLV-N is very closely related to orchid fleck virus, which typically infects *Cymbidium* species.

## GENOME ANNOUNCEMENT

Citrus leprosis virus cytoplasmic (CiLV-C) and nuclear (CiLV-N) types cause citrus leprosis in South and Central America. Both types of CiLV are transmitted by the Tenuipalpidae (false spider mites) *Brevipalpus* spp. (1). CiLV-C is a member of the genus *Cilevirus*, whereas CiLV-N is an unclassified *Rhabdovirus* with no prior sequence information available. CiLV-N was reported from the states of São Paulo, Rio Grande do Sul, and Minas Gerais in Brazil and Boquete in Panama (2). Citrus leprosis probably caused by CiLV-N used to be a major problem in Florida but has not been found there since the 1960s (3, 4). Leprosis-affected samples collected from different locations in Mexico were shipped to the USDA-APHIS-CPHST (the Center for Plant Health Science and Technology) lab in Beltsville, MD, for serological and molecular testing. Mexican leprosis-affected citrus samples from the state of Querétaro tested negative for CiLV-C and the recently discovered CiLV-C type 2 (CiLV-C2) (5) in enzyme-linked immunosorbent assay (ELISA) and reverse transcription-PCR (RT-PCR) (5–7), respectively. However, utilizing transmission electron microscopy, bullet-shaped virions similar to the virions previously reported for CiLV-N were observed in the nuclei and cytoplasm of the infected leaf tissues (8). Therefore, small RNAs (sRNAs) from symptomatic infected tissue were sequenced using Illumina technology to determine the complete genome sequence of CiLV-N.

Bioinformatic methods (9) were used to assemble the sRNA sequence data into 13 contigs of RNA1 and 11 contigs of RNA2. Based on the sequences of the assembled contigs, primer pairs were designed to amplify overlapping cDNA fragments to complete the genome sequence of CiLV-N RNA1 (6,268 nucleotides [nt]) and RNA2 (5,847 nt). Both RNAs had 3′-terminal poly(A) tails. The size and structure of the CiLV-N genome closely resemble the genome organization of orchid fleck virus (OFV), except for having smaller 3′-untranslated regions (UTRs) in RNA1 (53 nt) and RNA2 (35 nt) excluding the poly(A) tail. RNA1 contains five open reading frames (ORFs). ORF1 encodes the nucleocapsid protein (N), whereas ORF5 contains glycoprotein (G) (10). ORF2, ORF3, and ORF4 encode the putative phosphoprotein (P), cell-to-cell movement protein, and matrix protein (M), respectively (11). CiLV-N RNA2 contains only one ORF, which encodes the RNA-dependent RNA polymerase (RdRp) replication module.

ORFs of CiLV-N RNA1 and RNA2 have 90 to 91% nucleotide and 93 to 98% amino acid sequence identities with OFV sequences (GenBank accession no. AB244417 and AB516442 for RNA1, AB244418 and AB516441 for RNA2), and thus CiLV-N appears to be a citrus strain of OFV. However, due to the lack of other members in the genus, nucleotide diversity-based demarcation of species is not possible. In consultation with the International Committee on Virus Taxonomy, we consider CiLV-N to be a second member of the unattached genus *Dichorhabdovirus*. This is the first report of the complete genome sequence of CiLV-N and also the first report of a *Rhabdovirus* sequence in citrus.

### Nucleotide sequence accession numbers.

The CiLV-N genome sequences have been deposited in GenBank as accession no. KF209275 (RNA1) and KF209276 (RNA2).
